# Integration of Sensory Quanta in Cuneate Nucleus Neurons *In Vivo*

**DOI:** 10.1371/journal.pone.0056630

**Published:** 2013-02-08

**Authors:** Fredrik Bengtsson, Romain Brasselet, Roland S. Johansson, Angelo Arleo, Henrik Jörntell

**Affiliations:** 1 Neural Basis of Sensorimotor Control, Department of Experimental Medical Science, Lund University, Lund, Sweden; 2 Unit of Neurobiology of Adaptive Processes, CNRS–University Pierre & Marie Curie, Paris, France; 3 Physiology section, Department of Integrative Medical Biology, Umeå University, Umeå, Sweden; Tokyo Medical and Dental University, Japan

## Abstract

Discriminative touch relies on afferent information carried to the central nervous system by action potentials (spikes) in ensembles of primary afferents bundled in peripheral nerves. These sensory quanta are first processed by the cuneate nucleus before the afferent information is transmitted to brain networks serving specific perceptual and sensorimotor functions. Here we report data on the integration of primary afferent synaptic inputs obtained with *in vivo* whole cell patch clamp recordings from the neurons of this nucleus. We find that the synaptic integration in individual cuneate neurons is dominated by 4–8 primary afferent inputs with large synaptic weights. In a simulation we show that the arrangement with a low number of primary afferent inputs can maximize transfer over the cuneate nucleus of information encoded in the spatiotemporal patterns of spikes generated when a human fingertip contact objects. Hence, the observed distributions of synaptic weights support high fidelity transfer of signals from ensembles of tactile afferents. Various anatomical estimates suggest that a cuneate neuron may receive hundreds of primary afferents rather than 4–8. Therefore, we discuss the possibility that adaptation of synaptic weight distribution, possibly involving silent synapses, may function to maximize information transfer in somatosensory pathways.

## Introduction

Tactile sensibility is critical for our interactions with the external world [1], [2]. When the hand interacts with objects, cutaneous mechanoreceptors transduce mechanical events at the skin into gradable receptor potentials in the peripheral endings of the afferent nerve fibers [3]. The receptor potential is encoded into action potentials, also termed spikes, which propagate the sensory information into the central nervous system [4]. Since spikes are all-or-none events, the smallest possible sensory entity, or the sensory quantum, is a single spike generated at the nerve ending.

Human microneurography, in which the spikes propagating in single afferent nerve fibers are recorded, has been instrumental in defining the functional organization and limitations of our peripheral somatosensory system [5], [6]. For example, the sensory input elicited by different types of primitive shapes contacted by the fingertips under various conditions has recently been characterized [7]–[9].

However, our understanding of how tactile afferent signals are processed by the central nervous system is still very limited. Since the afferent signals underlying discriminative touch are first processed in the cuneate nucleus [10], [11], a critical issue is to resolve how the quantal sensory information is processed at this primary node, in which hundreds of afferents have been suggested to make synaptic contact with each second order (‘relay’) neuron [10].

In the present account, we analysed how excitatory postsynaptic potentials (‘EPSPs’) generated by spikes in different primary afferents are integrated in cuneate neurons. Based on intracellular, whole cell patch clamp recordings in decerebrated cats, we show that few (4–8) primary afferents that have large synaptic weights (or efficacy) dominate the input to individual cuneate neurons. Furthermore, using computer simulations we found that this number of active synapses is compatible with optimal information transfer of tactile information represented in populations of human primary afferents during natural tactile stimulations.

## Results

**Figure 1:**
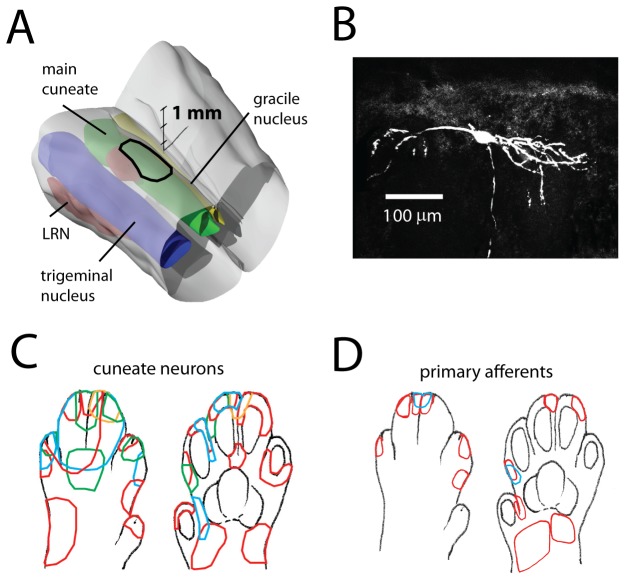
Recording area and receptive fields. **A**: Location of the main cuneate nucleus (green) in the lower brain stem. The region recorded from is outlined. A 3d scale bar is inserted. LRN, lateral reticular nucleus. **B**: Example of a cuneate neuron, displayed in the sagittal plane, that was recorded from and stained with neurobiotin. **C & D**: Receptive field outlines of the cuneate neurons and of the primary afferents recorded from in this study. Outlines are indicated in different colors for clarity.

We made patch clamp recordings from cuneate neurons (intracellular whole cell mode, N = 44) and primary afferents (loose cell-attached mode, N = 15) in non-anesthetized, adult cats, decerebrated at the collicular level. The neurons were all located in the dorsal half of the middle to rostral main cuneate nucleus (+/−2 mm in the rostrocaudal plane relative to the obex) (**Fig. 1A & B**), which has the highest representation of sensory input from the digits [12]–[14]. This part of the cuneate nucleus is believed to lack direct inputs from muscle afferents [15].

The resting membrane potential of the cuneate neurons was −52.8+/−1.0 mV (0 pA bias current; mean +/−1 s.d.; N = 42) and the membrane input resistance and time constant was 96+/−43 MOhm and 5.2+/−1.8 ms, respectively. In agreement with previous observations [16], the neurons fired spontaneously with ∼10 spikes/s in the absence of cutaneous stimulation. All neurons included in this study at least occasionally fired doublets or triplets of spikes (cf. [17]). All cuneate neurons and primary afferents were activated from skin hairs. Mapped with manually applied pointed skin stimulations, the area of the cutaneous receptive fields of the cuneate neurons ranged between 5 and 100 mm^2^ (20+/−17 mm^2^, N = 44) (**Fig. 1C**). The size of the receptive fields of the primary afferents, recorded in the entrance zone to the cuneate nucleus, ranged between 4 and 45 mm^2^ (12+/−10 mm^2^, N = 15) (**Fig. 1D**). The fact that the receptive fields of the cuneate neurons were larger than the receptive fields of the primary afferents (p<0.05, t-test) suggest convergence of synaptic inputs from multiple primary afferents on individual cuneate neurons, although the sample was too small to allow a conclusive, more systematic comparison (see also [18]).

**Figure 2:**
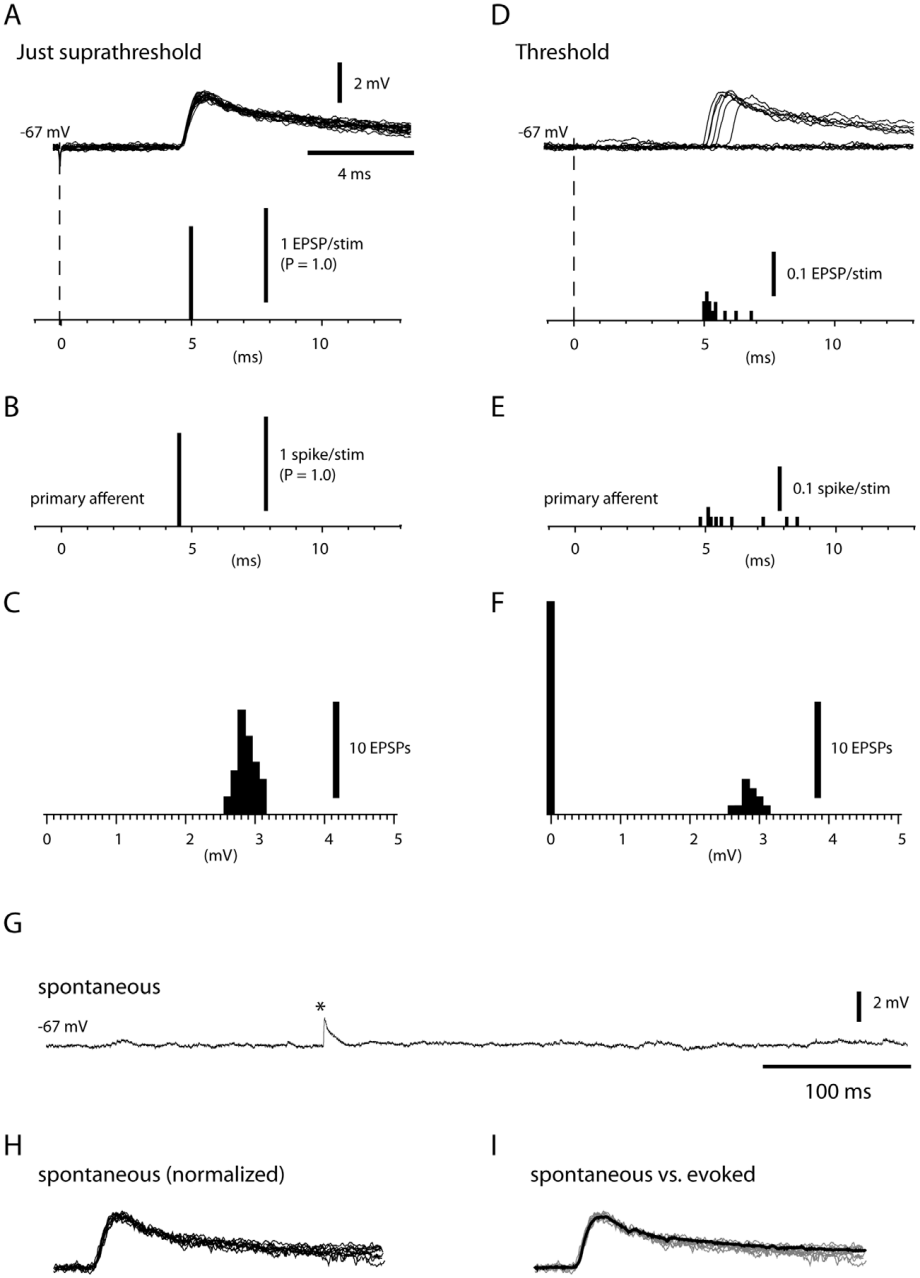
Synaptic responses. **A**: Superimposed, consecutive, unitary EPSPs evoked by electrical skin stimulation by just suprathreshold stimulation in the receptive field. Histogram at bottom (bin width = 0.1 ms) illustrate the non-variable response latency times. Every stimulation evoked an EPSP at the same response latency time (i.e. the firing probability at this time was 1.0). Dashed line indicates time of stimulation in both (A) and (D). **B**: For comparison, a corresponding histogram of the response latency times of a primary afferent spike using just suprathreshold stimulation intensity. **C**: Histogram of EPSP amplitudes evoked at just suprathreshold stimulation intensities (same cell as in (A)). **D**: Superimposed, consecutive responses evoked by threshold electrical stimulation in the receptive field (N = 12 stimulations). Only 6 of these stimulations evoked EPSPs, which had variable response latency times but essentially non-variable amplitudes (‘all-or-none’). The dashed vertical line indicates time of stimulation. Histogram at bottom illustrates the response latency times of the 13 EPSP responses obtained over 50 stimulations. Same calibrations as in (A). **E**: Response latency times of a primary afferent spike at threshold stimulation intensity. Note qualitative similarity with the spread of response latency times for the EPSP, as opposed to the non-variable latency times in (A,B). **F**: Amplitudes of EPSPs with a response latency of 5–7 ms evoked in the same cuneate neuron as represented in (D) by repeated near-threshold stimuli of constant intensity. Note the all-or-none character of the evoked response. **G**: Spontaneous activity was typically very low, with occasional spontaneous EPSPs (asterisk). **H**: Superimposition of spontaneous EPSPs with similar peak amplitudes as the evoked EPSP in (A, C) from the same cell. The peak amplitudes of the EPSPs were normalized. **I**: Comparison of raw spontaneous EPSP (grey traces) with an average of evoked EPSPs (black trace). Note the near complete congruence between spontaneous and evoked EPSPs. (H,I) same calibrations as in (A).

We investigated synaptic activity in cuneate neurons evoked by spikes in single primary afferents by applying weak electrical stimuli through needles inserted superficially in the skin within the neurons' mechanically defined receptive field. To eliminate spiking activity in the cuneate neuron, we hyperpolarized its resting membrane potential to ∼−67 mV with current injection. Stimuli of intensities just above the threshold for evoking a synaptic response ('suprathreshold', see below) consistently elicited an excitatory postsynaptic potential (1.00+/−0.00 EPSPs per stimulation, tested for 5 cuneate neurons) (**Fig. 2A**) with a consistent peak amplitude (coefficient of variation (CV) = 0.07+/−0.012) (**Fig. 2C**). Also the primary afferents responded with a similar high degree of fidelity at suprathreshold stimulation (1.00+/−0.00 spikes per stimulation, N = 15) (**Fig. 2B**). For both primary afferent spikes and primary afferent EPSPs, the variability of the unitary response latency time was extremely low (CV = 0+/−0% for both populations, calculated with a time resolution of 0.1 ms) (**Fig. 2A,B**). The difference between the overall response latency times of evoked EPSPs (5.1+/−0.5 ms, N = 22, evoked EPSPs at both suprathreshold and threshold intensities, see below) and evoked primary afferent spikes recorded at the entrance zone to the cuneate nucleus (4.5+/−0.4 ms, N = 15) allowed for, on average, a 0.6 ms synaptic delay.

Using a gradually increased electrical skin stimulation, single recruited primary afferent synaptic inputs were identified by the all-or-none activation of their excitatory postsynaptic potentials (EPSPs) as the stimulation exceeded the threshold for that synaptic input (**Fig. 2D,F**). In agreement with numerous previous, related neurophysiological investigations, this all-or-none characteristic of the response was an indication that the evoked EPSPs originated from the spiking activity in a single presynaptic afferent [19]–[22], for which the stimulus was at threshold strength eliciting all-or-none-responses in the afferent axon. Accordingly, the variability across test stimuli regarding the occurrence of responses and the response latencies of evoked EPSPs in cuneate neurons, matched the corresponding variability observed for single primary afferents when stimulated at intensities close to firing threshold (**Fig. 2E**). The qualitatively similar jitter in the response latency times for both EPSPs and primary afferent spikes (**Fig. 2D,E**), which was not present at just suprathreshold intensity (**Fig. 2A,B**), could readily be explained by that the electrical skin stimulation just barely reaches firing threshold in the mechanoreceptor of the primary afferent [3]. For 16 cuneate neurons analyzed in this respect, the peak amplitudes of reliably detected, unitary (all-or-none) EPSPs ranged between 1.2 and 6.9 mV (3.7+/−1.5 mV) and the coefficient of variation (CV) estimated over 15–40 successive responses was 0.08+/−0.009. The average rise time of these EPSPs, defined as the time between the points corresponding to 10%–90% of the peak amplitude, was 1.08+/−0.15 ms and half-decay times 6.4+/−1.9 ms (N = 16).

Without electrical stimulation, the background membrane potential was often quiet with the occurrence of occasional spontaneous EPSP-like events (**Fig. 2G**) (measured to 0.69+/−0.36 Hz in N = 15 cells with sufficiently long spontaneous activity recorded at about −67 mV). If the receptive field was partly located on the ventral side of the paw, which was in contact with the support against which the paw rested, the rate of spontaneous EPSP-like events was typically somewhat higher than otherwise. Assuming that these events could reflect synaptic responses evoked by spontaneous activity in the primary afferents (which were spontaneously active at 0.05+/−0.33 Hz, N = 15), and thus could be used as an indication that the evoked unitary EPSPs represented the activity of a single primary afferent synapse, we compared the time course of these events (**Fig. 2H**) with unitary EPSPs evoked by threshold stimulation. As shown in **Fig. 2I**, the spontaneous EPSPs (superimposed after normalization to the peak amplitude, see Methods) had a very similar time course to that of the unitary evoked EPSPs. In order to quantify this observation, we calculated RMS values for the evoked EPSPs and for the spontaneous EPSPs, in both cases after subtracting the average response of the evoked EPSP. For the cell illustrated in **Fig. 2**, the RMS values for the evoked EPSPs were 0.13+/−0.07 mV, whereas for the spontaneous EPSP the RMS value was 0.14+/−0.07. These values were not significantly different (P = 0.65, Student's T test). A similar analysis could be made for 6 other cells – in all cases, the spontaneous EPSPs were congruent with the time course of the unitary evoked EPSP (the P-values for the statistical comparisons were 0.47–0.69). This indicated that the time course of spontaneous and unitary evoked EPSPs was highly similar, strongly suggesting that the evoked unitary EPSPs represented the input from a single primary afferent.

**Figure 3:**
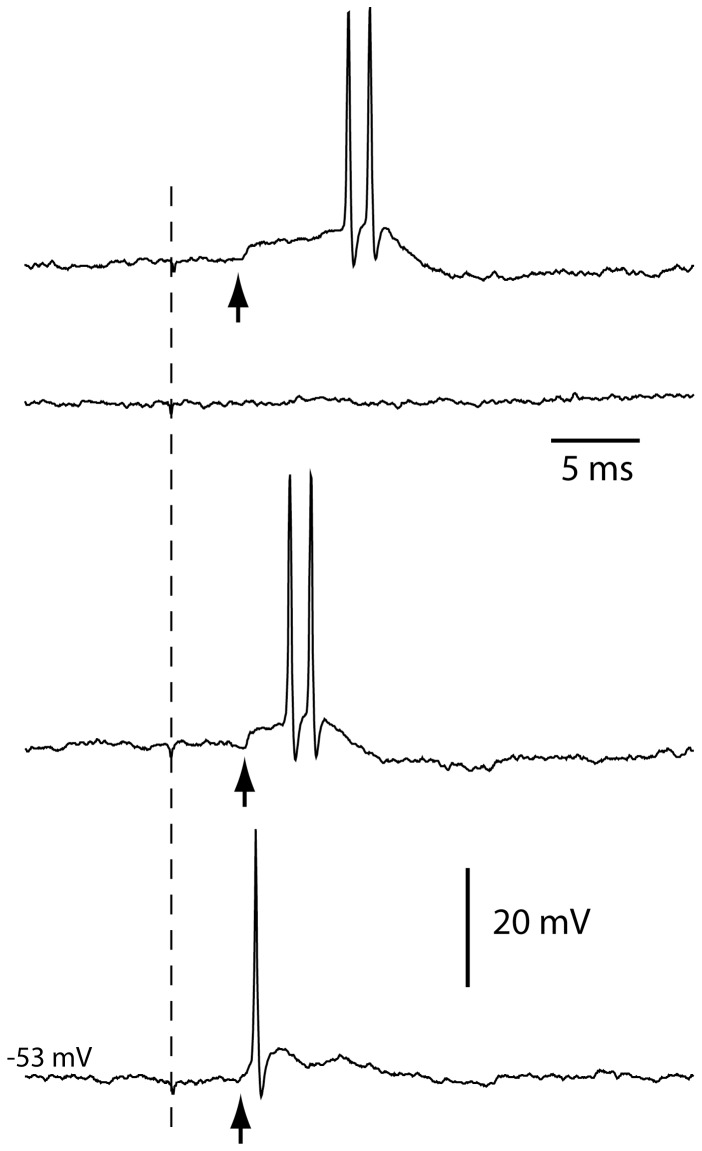
Electrical skin stimulation at threshold intensity in cuneate neuron at rest. In order to explore the EPSP-to-spike coupling in the cuneate neuron, the recording was made without hyperpolarization, i.e. the neuron was recorded at its normal resting potential. Threshold electrical skin stimulation evoked all-or-none EPSPs, i.e. they sometimes occurred (arrows) and sometimes not (second trace from the top). When the EPSP did occur, the probability that it was followed by a spike was high (see text).

When the neurons were recorded at rest, without the artificial hyperpolarization applied to explore the synaptic inputs, threshold EPSPs exhibited a high EPSP-to-spike coupling (98+/−0.7% mean+/−sem, analyzed for 5 neurons, 20 stimulations each), although with a response latency time of the spike that could vary greatly (**Fig. 3**). This finding was important since it indicated that the receptive fields delineated for the spike responses (**Fig. 1**) are valid also for the primary afferent synaptic input, i.e. it is unlikely that there would be primary afferent synaptic inputs from outside the receptive field defined for the spike responses. Since the neurons were spontaneously active at 10 Hz, despite a much lower frequency of spontaneous EPSPs (see above), the membrane potential is normally located above firing threshold and any small depolarization from a primary afferent synapse (or elsewhere) would increase the firing probability and would hence not pass undetected.

**Figure 4:**
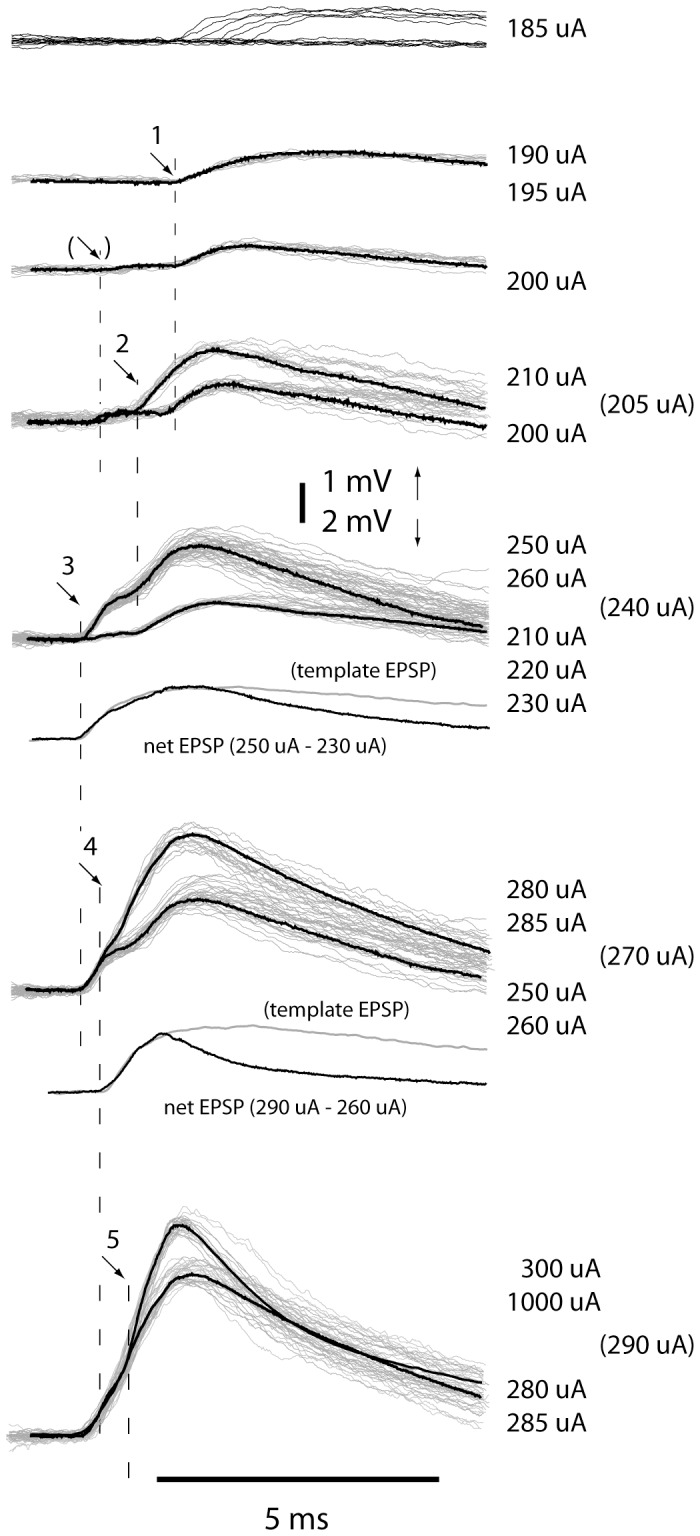
Recruitment of EPSPs during a graded increase in the intensity of the electrical skin stimulation. Superimposed raw traces (grey) and averaged responses (black) for stimulation intensities indicated to the right. Multiple stimulation intensities indicated to the right of a trace panel indicate that the averaged responses evoked by the different intensities were of the same amplitude. In panels with two different averaged responses overlayed, the top stimulation intensities correspond to the top trace and vice versa. This illustrates the stepwise changes in the responses obtained to a graded increase in stimulation intensity. At stimulation intensities indicated in brackets to the right, all-or-none responses that straddled between the levels of response amplitudes of the two averaged traces were evoked. Insets indicated by ‘net EPSP’ represent the net response added at the higher stimulation intensity (obtained by subtracting the average response obtained at the lower stimulation intensity from the average response obtained at the higher intensity). The net EPSP is overlayed with a scaled template EPSP obtained from the same neuron (grey). Vertical dashed lines indicate the average response latency time of the EPSPs recruited at the given stimulation intensity. (Arrow in brackets indicates possible EPSP component, but with a peak amplitude that was too low to be analyzed at the unitary level).

Collectively, these results showed that the latency times and peak amplitudes of primary afferent synaptic responses evoked by electrical skin stimulation had a low variability and that these synapses could have a high efficacy, as reflected in the large EPSP amplitudes. To obtain an estimate of the number of afferents providing significant synaptic inputs to individual cuneate neurons, we analyzed the recruitment of synaptic inputs in response to a gradual increase of the intensity of the cutaneous electrical stimulation. As the stimulation intensity was gradually increased from threshold, EPSPs were recruited in a step-wise fashion until the response saturated in a large, compound EPSP (**Fig. 4**). Apart from being recruited according to the all-or-none principle and having the same waveshape as a template unitary EPSP recorded in the same respective cell, each recruited EPSP also displayed a relatively fixed response latency time at just above its stimulation threshold, in a similar fashion as illustrated in **Fig. 2A** **(** **Fig. 4** **)**. For most unitary EPSPs (72% of the unitary EPSPs in 16 neurons), we were able to determine the response onset latency time at just suprathreshold stimulation intensity and to compare it with the response latency time of the ‘previous’ unitary EPSP, recruited at a lower stimulation intensity. In these cases, the response latency times of the ‘newly’ recruited EPSP were statistically different from those of the ‘previously’ recruited EPSP at p<0.01 in 97% of the comparisons. These three independent measures (all-or-none recruitment, template matching and specific response latency times) together provide evidence that the recruited EPSPs really represented unitary EPSPs, corresponding to the synaptic response of a single primary afferent.

The peak amplitude of the saturated compound EPSP was 14.9+/−0.3 mV (N = 16) (**Fig. 4**). Based on the compound EPSPs recorded in the 16 neurons we estimated the number of afferents that contributed with unequivocally identified unitary EPSPs (see Materials and Methods) to be between 2 and 8 (mean: 3.7+/−0.4). The peak amplitudes of these EPSPs ranged between 1.2 and 7.0 mV (mean: 3.8+/−1.5 mV). A summation of the peak amplitudes of the identified unitary EPSPs for each neuron rendered values (10.3+/−2.4 mV) that corresponded to 69+/−16% of the amplitude of the maximal compound EPSP (14.9+/−0.3 mV) evoked by cutaneous stimulation at supramaximal intensity. Given these results, and that in cases of less than 4 unequivocally identified unitary EPSPs we estimated that there were in addition at least 2 apparent EPSP units contributing to the compound EPSP, we estimated that spikes in 4–8 primary afferents accounted for the saturation of the maximal compound EPSP.

**Figure 5:**
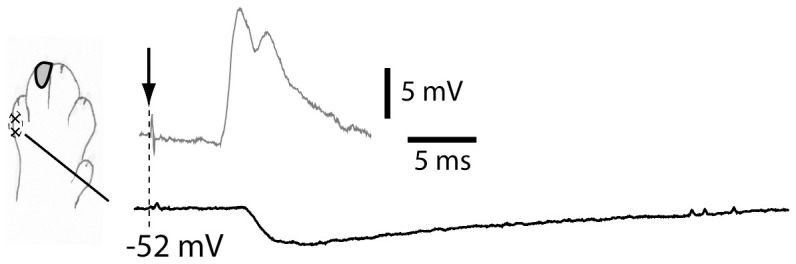
Inhibitory postsynaptic potentials (IPSPs) evoked by electrical cutaneous stimulation. Solid black trace shows IPSP (averaged) evoked in a cuneate neuron by electrical cutaneous stimulation applied through a pair of electrodes located in the skin (x–x) outside the neuron's cutaneous receptive field (outlined zone). The membrane potential of the neuron was held at −53 mV. Maximal evoked average EPSP (evoked inside the receptive field) is shown in grey to illustratethe temporal relationship between evoked EPSPs and IPSPs. The EPSP was recorded at a membrane potential of −67 mV to prevent spiking. The longer latency of the IPSP indicates an indirect, disynaptic activation, due to the extra synaptic relay over inhibitory interneurons.

The saturated compound EPSPs was reached already at a stimulation intensity of 1.95+/−0.22 times the threshold intensity. The rather narrow intensity range for the graded response suggested that the primary afferents exciting the cuneate neurons essentially terminated within their mechanically delineated receptive fields. We verified this by showing that strong electrical skin stimulation applied at various distances (3–15 mm) from the borders of the delineated receptive fields failed to evoke EPSPs within the appropriate time window (5–10 ms after the skin stimulation); 1–5 sites were tested for 16 neurons at ∼20 times the average threshold for eliciting responses in primary afferents. However, such stimuli could evoke inhibitory postsynaptic potentials (IPSPs) (**Fig. 5**). In agreement with previous findings, the response latencies of these IPSPs were longer than the latencies observed for EPSPs [23]. Synaptic inhibition was not the focus of the present work, but has previously been investigated [24].

**Figure 6:**
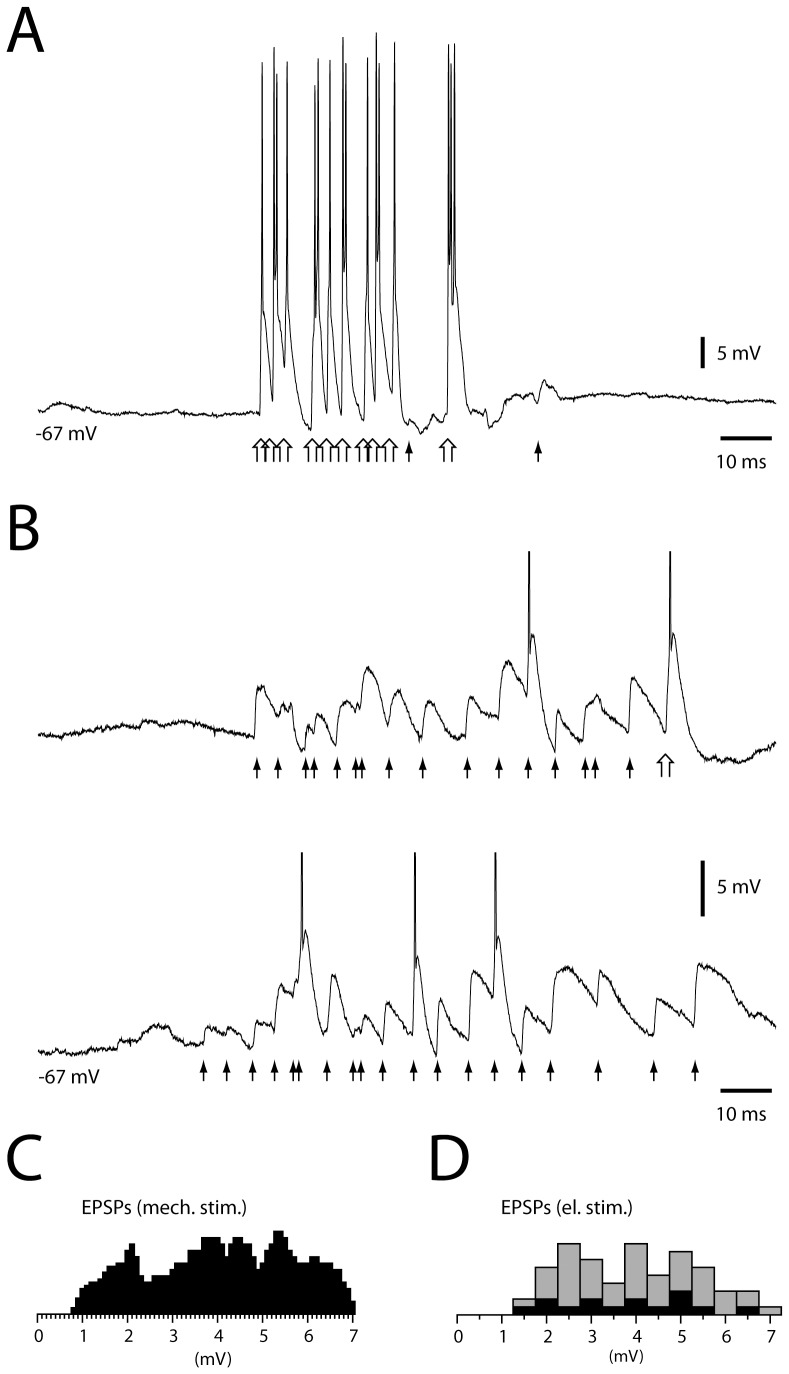
EPSP responses evoked by cutaneous stimulation. **A**: Response evoked by light, mechanical stimulation normally used for receptive field mapping. Filled arrows indicate onsets of presumed unitary EPSPs and empty arrows indicate compound EPSPs, defined as depolarizing events of more than 10 mV within 1 ms, presumably generated by multiple unitary EPSPs activated nearly simultaneously. **B**: In the same cell, responses evoked by very light mechanical skin stimulation. The amplitudes of action potentials are truncated. Note difference in voltage scale compared to (A). **C**: Histogram of the peak amplitudes of EPSPs evoked by 5–10 epochs of stimulation, for all cells explored with very light skin stimulation. **D**: For comparison, histogram of the amplitudes of EPSPs evoked at threshold electrical skin stimulation (black bars) and of unitary EPSPs evoked by suprathreshold electrical skin stimulation (grey bars) (cf. Figure 4).

We also analyzed excitatory events in cuneate neurons in response to mechanical skin stimulation. The manually delivered tactile stimuli used to outline the receptive field of the neurons typically evoked intense compound EPSPs and spiking activity that made it difficult to identify unitary EPSPs (**Fig. 6A**). However, using very light tactile stimuli and negative bias current to reduce the spiking output, in 11 neurons we could distinguish unitary EPSPs (**Fig. 6B**). Even though the complexity of the synaptic responses made it difficult to measure precisely the amplitude of the identified EPSPs, the distribution of the measured EPSP amplitudes was similar to that of unitary EPSPs elicited by electrical cutaneous stimulation (**Fig. 6C & D**).

**Figure 7:**
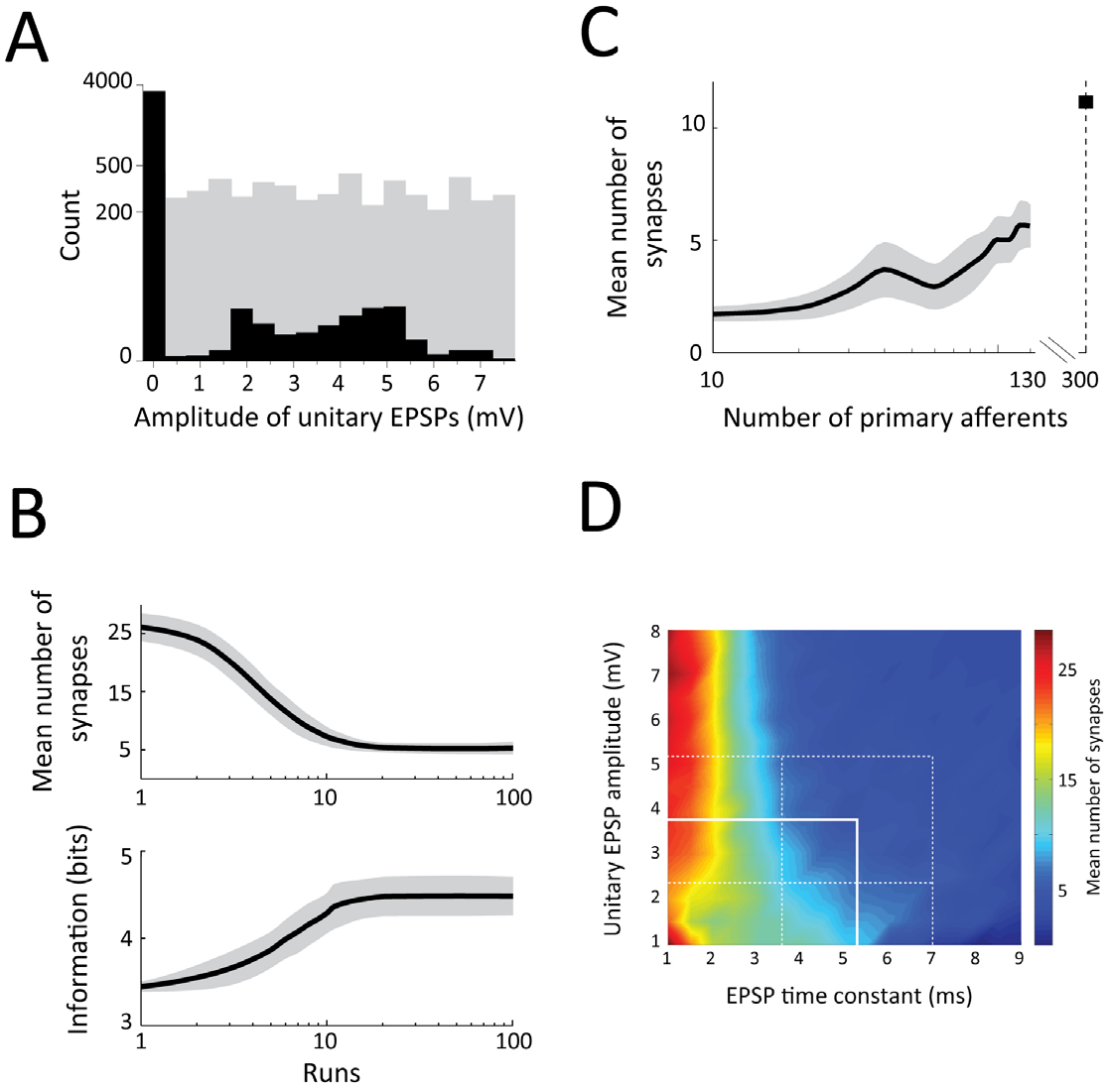
Simulation of the relationship between distribution of synaptic efficacy and information transfer in cuneate neurons. A: Histogram showing EPSP amplitude distribution for N = 100 simulated neurons with 130 primary afferent synapses each. The random EPSP distribution (grey) and specific EPSP distribution (black), the latter reproducing to the experimentally observed amplitude distribution (Fig. 6C), corresponded to the start and end points, respectively, of the simulation (runs 1–100). B: Relationship between number of synapses and information transfer. Top: Starting from synaptic weights drawn from random (uniform distribution; grey distribution in (A)) the number of synapses decreased significantly (p<0.01, ANOVA test) as the result of a gradient-based information maximization algorithm, rendering the weight distribution of primary afferent synapses from random to bimodal (with many synapses eliminated or having 0 synaptic efficacy) (black distribution in (A); Hartigan test, dip = 0.0275, p<0.001). Bottom: The mutual information between cuneate responses and primary inputs increased significantly (p<0.01, ANOVA test) with the number of synapses becoming virtually silent regarding EPSP effects. In both cases, the graphs display the mean value +/−s.d. C: Relationship between the number of primary afferents and the number of synapses required to achieve maximal information transfer. The value for 300 primary afferents was not measured but projected from the data using linear regression. D: Relationship between the maximal unitary EPSP peak amplitude, the EPSP time constant, and the mean number of synapses required to achieve maximal information transfer in the simulated cuneate neurons. White solid lines indicate mean values for the cuneate neurons recorded in the present study, white dashed lines indicate the standard deviation of these values.

Given that a cuneate neuron is considered to receive inputs from about 300 primary afferents [10], it was surprising that only a few (4–8) afferents appeared to functionally dominate the input. By a modeling approach, we sought the distribution of synaptic efficacy associated with maximum information transfer in the primary afferent-to-cuneate neuron synaptic relay. We simulated N = 100 independent cuneate neurons by using a stochastic integrate-and-fire neuronal model [25] parameterized based on the electrophysiological data obtained in our neuronal recordings (see Materials and Methods). Each simulated cuneate neuron received as inputs the spike trains recorded from up to 130 single primary tactile afferents innervating the human fingertip in response to 81 distinct natural tactile stimuli (see [7], [9]). During each simulation run, each single cuneate neuron received 100 presentations of the population of primary afferent responses to the entire set of stimuli. A gradient-based information maximization algorithm [26] shaped the distribution of synaptic efficacies of primary afferent-to-cuneate connections and, consequently, the distribution of unitary EPSP amplitudes. In the first run, we employed a uniform random distribution of synaptic weights (gray bars in histogram, **Fig. 7A**). During the operation of the gradient-based information maximization algorithm over consecutive runs, the mutual information [27] between cuneate responses and primary afferent inputs increased significantly (p<0.01, ANOVA) with a significant decrease (p<0.01, ANOVA) in the number of synapses showing high weight (**Fig. 7B**). When near maximal information transmission was achieved after some 20 runs, only very few (less than 10) synapses provided synaptic input. The amplitude distribution of the EPSPs measured in the 100 simulated cuneate neurons after convergence of the algorithm (black bars in histogram, **Fig. 7A**) was strikingly similar to that experimentally observed (**Fig. 6C & D**).

In the simulation, we also addressed the relationship between the number of primary afferents available and the number of synapses required to achieve maximal information transfer. The outcome indicated that a higher number of afferents requires a higher number of synapses to achieve maximal information transfer (**Fig. 7C**). A projection of these data up to 300 primary afferents, which is a coarse estimate of the number of primary afferent synapses per cuneate neuron [10], suggested that information transfer was optimized with less than 10 synapses showing high weight (**Fig. 7C**). Finally, our simulations indicated that the combination of EPSP amplitudes and time constants that we observed experimentally tended to minimize the number of synapses required for effective information transfer (**Fig. 7D**).

## Discussion

The central advance of this study is the finding that spikes in a limited number of single primary afferents innervating a relatively small cutaneous area elicited large EPSPs in cuneate neurons. The observed synaptic weight distribution is found to result in a high rate of information transfer for the patterns of primary afferent spike trains that can be generated during touch. Viewed against the background of known anatomical relationships between primary afferents and cuneate neurons, these findings have several potential implications for the arrangement of primary afferent synaptic inputs to the neurons of the cuneate nucleus.

A prerequisite for the present analysis of primary afferent synaptic inputs was that the EPSPs generated by electrical skin stimulation exhibited essentially non-fluctuating peak amplitudes and response latency times (**Fig. 2A,B**). Likewise, even though the probability of EPSP responses decreased when evoked by threshold stimulation and the variability in response latency times increased, the peak amplitude of evoked EPSPs was consistent (**Fig. 2**). Combined with the congruence in the time course demonstrated for the spontaneous and evoked unitary EPSPs (**Fig. 2D**–**I**), the electrical skin stimulation used was shown to most probably being capable of activating single primary afferents in a controlled fashion. This consistency of evoked EPSP responses allowed us to assess the recruitment of unitary EPSPs as the stimulation intensity was increased from threshold (**Fig. 2D,E**) to supramaximal levels (**Fig. 4**) in a similar fashion as synaptic inputs has been previously analyzed for other systems *in vitro* [19]–[22], [28].

Previous studies of the neurons of the cuneate nucleus have suggested a strong coupling between single primary afferents and cuneate neuron discharge [29]–[31]. Given that we observed that the cuneate neurons fired spontaneously despite a very low spontaneous EPSP frequency, the resting potentials of these neurons are probably normally above firing threshold. This would provide a natural explanation for the above findings – if the neurons are already above firing threshold, any small depolarization will increase the probability of firing. However, supposedly the cuneate neurons do something more than just acting as passive relays of information from single primary afferents. For this, they would need to integrate information from more than one primary afferent and one of the aims of the present paper was to characterize the nature of the primary inputs integrated. Our analysis indicated that the neurons located in the dorsal part of the cuneate nucleus concerned with hair follicle input integrate information from 4–8 primary afferents, which are activated from a very small part of the skin. Moreover, it also indicated that the synaptic weights of the individual inputs is specific and can be highly segregated so that some primary afferents can have a larger weight than others.

Primary afferents run dorsally along the rostro-caudally oriented cuneate nucleus, issuing numerous axon collaterals along their course [14], [32]. Afferents innervating the different digits are organized in a longitudinal, columnar-like, somatotopy, in the dorsal part of the nucleus [12]–[14], [32], which is considered to receive primary afferent inputs only from the skin [15]. The number of primary afferents converging on a single cuneate neuron has not been measured directly. However, different types of estimates suggest that the number should be well above 100. First, based on the areal size and density of the terminal arborizations of single primary afferents, the number of overlapping primary afferents at any point in the cuneate nucleus was estimated to be about 300 [10], [14]. A second estimate can be derived from the observation that each primary afferent fiber gives off 1,700 presynaptic terminals distributed over 6–15 collateral branches given off along the rostrocaudal course of the stem axon [10], [14] and that there are about 40,000 primary afferents entering the cat cuneate nucleus [14], [33], [34]. Combined with the estimate of 76,000 neurons in the cat cuneate nucleus [35], the second estimate would predict that there are nearly 900 primary afferent synapses per cuneate neuron. A third estimate comes from morphological observations of cuneate neurons. Cuneate neurons have 6–7 dendrites which extend at least 100–200 um each (Fig. 1B, [36]). Assuming an average dendritic diameter of 4 um [37] the total membrane surface area would amount to 7,500–15,000 sq.um. Since a synaptic bouton on average occupies 7 sq.um [14], there is room for 1,000–2,000 synapses on the dendrites of each cuneate neuron. Not all of these have to be primary afferent synapses, but since it is a main source of synaptic input, it seems reasonable to conclude that it is unlikely that there are fewer than 100 primary afferent synapses per neuron. These organizational and numerical considerations suggest that the cuneate neurons should have had larger cutaneous receptive fields than we observed, possibly receiving excitatory inputs from afferents distributed all over the digit. However, electrical skin stimulation outside the manually defined receptive fields, typically covering just a small part of a digit, invariably failed to evoke EPSPs (but often evoked IPSPs, **Fig. 5**). In addition, all neurons were routinely checked for inputs from the entire forelimb in the non-hyperpolarized state, in which cuneate neurons are highly responsive to peripheral input [16]. Altogether, the simplest explanation for the discrepancy between the number of primary afferent we have identified generating EPSPs in cuneate neurons and the number of afferents expected to provide such inputs based on the anatomical studies above is that a large proportion of the existing synapses are functionally silent. An alternative possibility, which however do not seem to be supported by the relatively wide extent of the transverse distribution of the terminal arborizations for each individual axon collateral of the primary afferents [14], is that a majority of the synaptic terminals of a single axon collateral is making synaptic contact on a single cuneate neuron. In that case, the effective primary afferent synapses that we observed would be accounted for by 4–8 primary afferents each making about a 100 synapses per neuron. However, since multiple anatomically separable synaptic contacts formed by a single afferent on the same target neurons is a relatively rare feature in the central nervous system, and then typically is confined to a maximum of 10–15 contacts per afferent in immature animal [38], this possibility seems unlikely as an exhaustive explanation of our results.

The concordance of the presented neurophysiological and simulation results suggests that one potential function of the specific synaptic weight distribution may be to enhance the transfer of information represented in ensembles of primary afferents during natural tactile stimulation to other brain areas. This quantitative view, based on the infomax principle [39], [40], leads to more qualitative interpretations akin to the redundancy reduction principle in neural processing [41]–[43]. For example, the strengthening of a few synapses and the silencing of the rest might result from a developmental process mediating the formation of compact cuneate receptive fields. This possibility could be seen as a specific solution subsumed by the information maximization principle. In terms of information processing, large synaptic weights allow for a very reliable coupling between an afferent spike and a cuneate spike. However, in order for the cuneate neuron to not work continuously at a saturated firing rate, which would correspond to a low information transfer, only a small subset of afferents should be able to influence its dynamics. A few large weights thus seem optimal to convey information.

Assuming that the existence of multiple silent synapses on cuneate neurons is the most probable interpretation of our observations, it is interesting to compare the potential functional role of silent synapses in the cuneate as opposed to elsewhere in the brain. Silent synapses, as they have been described in other brain areas, can be turned effective through long-term potentiation (LTP) [44]–[46]. Immediate uncovering of previously silent synapses has been postulated as a major contributor to short-term plasticity of somatosensory pathways resulting in substantial changes of receptive fields of central neurons after limited peripheral denervation [10], [47]–[49]. Since more than 1000 cuneate neurons may receive synaptic inputs from a single primary afferent [14] there would be a substantial potential for functional reorganization in the cuneate nucleus. Uncovering of silent synapses in the cuneate neurons may be critical for upholding discriminative touch during the profound changes in the structural and functional features of the peripheral nervous system that occur with normal aging and after injury [50], as well as for acquiring new sensorimotor skills.

## Materials and Methods

### Ethics statement

The experimental procedures were approved in advance by the Malmö/Lund Animal Research Ethics Committee (permit number and approval-ID: M32-09).

### Preparation

Adult cats were initially deeply anesthetized with propofol (Diprivan® Zeneca Ltd, Macclesfield Cheshire, UK; total dose 180–260 mg). After decerebration at the intercollicular level, the anesthesia was discontinued (similar to [16], [51], [52]). The end-expiratory CO2, blood pressure and rectal temperature of the artificially ventilated animals were continuously monitored and maintained within physiological limits. To monitor the level of anesthesia before derebration, we continuously measured the blood pressure and verified the absence of withdrawal reflexes to noxious stimulation. To monitor the state of the preparation after the decerebration, we in addition made EEG recordings from intact parts of the neocortex. EEG recordings were characterized by a 1–4 Hz oscillatory activity that was periodically interrupted by large-amplitude 7–14 Hz spindle oscillations lasting for 0.5 s or more. Such EEG patterns are normally associated with deep stages of sleep [53]. The EEG activity and the blood pressure remained stable, also on noxious stimulation, throughout the experiments. Mounting in a stereotaxic frame, drainage of cerebrospinal fluid, pneumothorax and clamping the spinal processes of a few cervical and lumbar vertebral bodies served to increase the mechanical stability of the preparation.

### Recordings and stimulation

The cuneate nucleus was accessed after the dura between the first cervical vertebrae and the skull had been cut and the extent of the nucleus had been electrophysiologically mapped. Patch clamp recordings were made from a zone ±2 mm in the rostrocaudal plane relative to the obex using pipettes pulled to 6–14 MOhm (Sutter micropipette puller P-97, Sutter Instruments Co., USA) and filled with a potassium-gluconate based solution (in mM: 145 K-gluconate, 10 HEPES, 7 KCL, 1 EGTA, 2 Mg-ATP, 0.1 CaCl(2); titrated to pH 7.35–7.4 using KOH) [51]. In total, we established recordings in the intracellular whole cell mode from 42 cuneate neurons. However, because of limited duration of the recordings, each of the analyses performed were based on a subset of these neurons as specified in the main text. Spikes in 15 primary afferents were recorded using the loose cell-attached mode. After qualitatively verifying that the neuron recorded was activated by skin input but not deep input (i.e. squeezing and muscle stretch), we outlined the cutaneous receptive fields of cuneate and primary afferent neurons using a fine-tipped blunt probe for gentle manual stimulation. For recording of the time-course and magnitude of the forces applied, the probe was attached to a strain gauge and mounted on the investigators index finger [16], [51]. For electrical skin stimulation we used square-wave pulses of 0.1 ms duration delivered at 1Hz through a pair of fine stainless steel needles (diameter  = 0.1 mm) inserted with 2–4 mm spacing superficially into the dermis in the center of the mechanically outlined receptive field.

Patch clamp pipettes were lowered into the brainstem under 3–10 times atmosphere pressure, which was reduced when the tip reached the dorsal part of the nucleus based on stereotactic and depth coordinates previously established with metal electrodes. At occasions of distinct increases in tip resistance when the electrode was advanced, the pressure was removed and a seal formation was attempted. Once obtained (0.5–7 GOhm), rapid application of negative pressure was used to gain access to intracellular space. For quality checks of the recordings, we monitored amplitudes of spikes (>25 mV required) and EPSPs evoked by skin stimulation. The resting membrane potential was defined as the average potential recorded between spikes at 0 pA bias current within 30 s after intracellular access (−50 to −55 mV). Whenever spike amplitudes or EPSP amplitudes had deteriorated by more than 20% from those initially recorded, the recording was stopped. Access resistance was 10–25 MOhm and compensated for off-line. Six neurons were stained by neurobiotin in the electrode solution. These neurons, all located in the cuneate nucleus, were studied in a confocal microscope (sagittal sections) and reconstructed morphologically from transparent z-stacks of contiguous confocal images.

### Data analysis

All data analysis was made off-line using in-house software. Unless otherwise indicated population estimates are given as **mean**±**1 SD**. Unpaired Student's t-tests were used for statistical testing. Coefficients of variation (CVs) were calculated based on 15–50 samples.

As in previous studies [16], [51], [52], we defined the onset of EPSPs after computing the standard deviation of the second time derivative of the membrane potential during a 10 ms interval that included baseline membrane activity starting 5 ms before the onset of the EPSP. The point when the recorded 2^nd^ time differential first exceeded a value greater than 2 standard deviations was taken as the start of a probable EPSP. Events in the membrane potential that occurred ≥5 ms after a preceding EPSP or IPSP-like event and had a time-course typical for EPSPs as assessed by template-matching (using scalable EPSP templates created from isolated unitary EPSPs from the same recorded neuron using in-house software, see [52]) were classified as EPSPs.

The amplitude of an EPSP was measured as the difference between the average membrane potential during a ±0.5 ms epoch around its peak amplitude and the average membrane potential during a 1 ms epoch preceding the onset of the EPSP. In the analysis of amplitudes of manually evoked EPSPs, we limited the analysis to EPSPs with amplitudes >0.6 mV since the recording noise prevented reliable measurements of smaller events. For analysis of EPSP components inside compound EPSP responses, identification of unitary EPSPs relied on template matching using a template generated based on spontaneously occurring EPSPs.

To obtain an estimate of the number of afferents providing synaptic inputs to individual cuneate neurons, we analyzed the recruitment of synaptic inputs in response to a gradual increase of the intensity of the cutaneous electrical stimulation. EPSPs considered recruited by electrical stimulation had to display all-or-none behavior at the recruitment threshold. To prevent inclusion of spontaneously occurring EPSPs, the onset of the candidate EPSP had to occur within <8 ms after the onset of the compound EPSP at least 3 times every 20 stimulations; due to the low rate of spontaneous activity in primary afferents, the probability that this would occur by chance was considered negligible. In the analysis of gradually recruited EPSPs with increasing stimulation intensity, only EPSPs that within 0.3 ms after their onset deviated in voltage by more than 3 s.d. from the responses obtained at the previous, lower stimulation intensity were used for statistical evaluation of latency time differences between unitary EPSPs (see text).

Spontaneous EPSPs were compared with all-or-none (unitary) EPSPs evoked from the skin at threshold stimulation intensity using calculations of the quadratic mean (or root mean square deviation from the average, RMS). First, the average of the evoked EPSPs was subtracted from the raw evoked EPSP responses. For each sample point, the deviation (in mV) from the baseline was measured and the value was squared. To minimize contamination from other events that could occur by chance, this value was calculated only for all sample values of the first 3 ms of the EPSP. Since the sample rate was 100 kHz, 300 values were obtained for each evoked response. The square root of the mean of these 300 values was taken as the RMS value of the EPSP response. The distribution of the quadratic means of the evoked EPSP responses was then compared to the RMS of the spontaneous EPSPs. However, the RMS values of the spontaneous EPSPs were not calculated against the average of the spontaneous EPSPs, but against the average of the evoked EPSP. In this way, a comparison between the two populations (unitary spontaneous vs. unitary evoked) of EPSPs could be done. In order to compensate for fluctuations in the amplitudes of the EPSPs, all EPSPs were first normalized with respect to their peak amplitude (calculated from an average of 20 adjacent sample values). To compensate for the change in the background noise resulting from the normalization, the obtained voltage was divided by the scaling factor needed to normalize the EPSP amplitude before the calculation of the RMS value.

### Neuronal modeling and information theoretical analysis

The shape of the experimentally observed unitary EPSPs was modeled by means of the following depolarization kernel function: 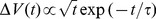
(1)with 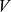
denoting the membrane potential, 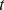 the time of arrival of an afferent spike and 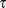  =  5 ms indicating the membrane time constant. The kernel function defined by Eq. 1 modeled the experimentally observed EPSP time-course better than more standard depolarization functions [25]. The peak amplitude of the EPSP function was normalized to 1 mV and then modulated by the synaptic efficacy (weight) 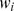 of the projection from each primary afferent 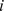, i.e. 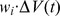. Multiple inputs added linearly to generate compound EPSPs:
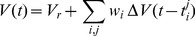
(2)where 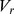  =  -53 mV is the resting membrane potential, 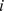 indicates primary afferents and 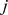 indexes the spikes conveyed by a primary afferent 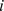 at times 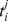. The probability of firing was computed, at each time *t*, according to:
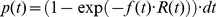
(3)where the function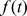 defines the instantaneous firing rate as: 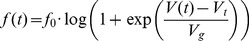
(4)with 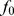 = 10 Hz denoting the spontaneous firing rate, 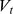 =  -50 mV the threshold potential, and 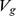 = 0.1 mV an escape noise factor [25]. The function 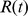 of Eq. 3 models the refractoriness property of the simulated neuron as a function of the time 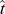 of the last emitted spike:
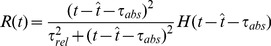
(5) where 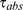 =  6 ms and 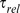  =  1 ms indicate the absolute and relative refractory period, respectively, and 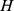 the Heaviside function.

All the simulations were carried out by using a time step 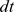 = 1 ms.

To assess the impact of synaptic efficacy distribution on the information transmitted by single cuneate neurons, as inputs we used a large data set of primary afferent responses to tactile stimuli of the human fingertip obtained through microneurography recordings from single afferents in the median nerve at mid-level of the upper arm [7]. Each single simulated cuneate neuron (N = 100) received synaptic input from ensembles of primary afferents responding to 81 distinct tactile stimuli applied to the fingertip with different combinations of force amplitudes, rates, directions and shape of stimulation surface. Though a primary afferent could make multiple synaptic contacts with a cuneate neuron, for the sake of simplicity we considered a single synaptic weight for each afferent that may be regarded as the aggregate efficacy of many small synapses. All these primary afferents had receptive fields on the stimulated fingertip. The entire spike train responses of all primary afferents were used as simultaneous inputs to the model cuneate neuron. The 81 distinct tactile stimuli belonged to a stimulus state space defined according to a set of four primary contact parameters: the curvature of stimulation surface (0; 100; 200 m^−1^), the magnitude of the applied force (1; 2; 4 N), the direction of the force with reference to the primary site of contact at the fingertip (ulnar, radial, distal, proximal, normal) and the angle of the relative force relative to the normal direction (5; 10; 20°). Data showing afferent responses to a subset of these stimuli (3 curvatures ×5 force directions) delivered at 4 N contact force and at 0 and 20° angle to the normal has previously been published [8], [9].

Shannon mutual information [27] was used to quantify the reliability of neurotransmission [54]. Mutual information 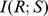 was measured between the set 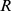 of responses of a single cuneate neuron and the set 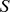 of primary afferent inputs. The entire spike temporal patterns of both outputs 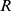 and inputs 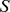 were encoded as binary words through a binning procedure [55], [56] (bin width = 1 ms). Shannon mutual information was computed as follows: 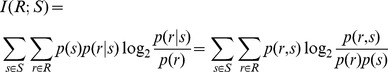
(6)where 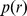 and 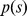 denote the marginal probability distributions for response and input spaces, respectively, 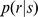 the conditional probability distribution, and 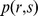 the joint distribution.

We ran a series of 13 simulations by varying the number of primary afferents (synapses) received by a single cuneate neuron from 10 to 130 (with increments of 10). During each simulation, every single cuneate neuron received 100 presentations of the primary afferent responses to the entire set of stimuli. We named the presentation of the entire stimulus set a ‘*run*’ (i.e. each simulation consisted of 100 runs).

At the beginning of each simulation, the efficacies of primary afferent-to-cuneate synapses were randomly initialized. Then, during each run, a gradient-based information maximization algorithm [26] was applied to shape the synaptic weight distribution online 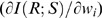 in order to optimize the capacity of single cuneate neurons to discriminate the 81 natural tactile stimuli. After each run, the mutual information corresponding to the current synaptic weight distribution was probed by presenting each primary afferent spatiotemporal pattern 100 times, which was sufficient to limit the sampling bias of mutual information estimation [57]. Each of the 13 simulations was repeated N = 100 times (to simulate 100 independent single cuneate neurons) and an ANOVA analysis was employed to quantify the statistical significance of the results (with *p*<0.01 considered as significant).
